# Mitochondrial DNA analyses revealed distinct lineages in an alpine mammal, Siberian ibex (*Capra sibirica*) in Xinjiang, China

**DOI:** 10.1002/ece3.10288

**Published:** 2023-08-02

**Authors:** Rui‐Rui Wang, Pei‐Pei Dong, Daisuke Hirata, Shamshidin Abduriyim

**Affiliations:** ^1^ College of Life Science Shihezi University Shihezi China; ^2^ Centre for Computational Biology Peter the Great Saint Petersburg Polytechnic University St. Petersburg Russia; ^3^ School of Life Science Peking University Beijing China; ^4^ Xinjiang Production and Construction Corps Key Laboratory of Oasis Town and Mountain‐Basin System Ecology Shihezi University Shihezi China

**Keywords:** *Capra sibirica*, divergence time, intraspecific relationships, mtDNA, Xinjiang of China

## Abstract

Maternal lineages of mitochondrial DNA (mtDNA) are recognized as important components of intra and interspecific biodiversity and help us to disclose the phylogeny and divergence times of many taxa. Species of the genus *Capra* are canonical mountain dwellers. Among these is the Siberian ibex (*Capra sibirica*), which is regarded as a relic species whose intraspecific classification has been controversial so far. We collected 58 samples in Xinjiang, China, and analyzed the mtDNA genes to shed light on the intraspecific relationships of the *C. sibirica* populations and estimate the divergence time. Intriguingly, we found that the mtDNA sequences of *C. sibirica* split into two main lineages in both phylogenetic and network analyses: the Southern lineage, sister to *Capra falconeri*, consisting of samples from Ulugqat, Kagilik (both in Xinjiang), India, and Tajikistan; and the Northern lineage further divided into four monophyletic clades A–D corresponding to their geographic origins. Samples from Urumqi, Sawan, and Arturk formed a distinct monophyletic clade C within the Northern lineage. The genetic distance between the *C. sibirica* clades ranges from 3.0 to 8.6%, with values of *F*_ST_ between 0.839 and 0.960, indicating notable genetic differentiation. The split of the genus *Capra* occurred approximately 6.75 Mya during the late Miocene. The Northern lineage diverged around 5.88 Mya, followed by the divergence of Clades A–D from 3.30 to 1.92 Mya during the late Pliocene and early Pleistocene. The radiation between the Southern lineage and *C. falconeri* occurred at 2.29 Mya during the early Pleistocene. Our results highlight the importance of extensive sampling when relating to genetic studies of alpine mammals and call for further genomic studies to draw definitive conclusions.

## Graphical abstract

Intriguingly, we found that the mtDNA sequences of *Capra sibirica* split into two main lineages in both phylogenetic and network analyses: the southern lineage, sister to *Capra falconeri*, and the northern lineage further divided into four monophyletic clades A–D corresponding to their geographic origins. The split of the genus Capra occurred approximately 6.75 Mya during the late Miocene. The northern lineage diverged around 5.88 Mya, following the divergence of Clades A–D from 3.3 to 1.9 Mya during the late Pliocene and early Pleistocene. The radiation between the southern lineage and *C. falconeri* occurred at 2.29 Mya during the early Pleistocene.

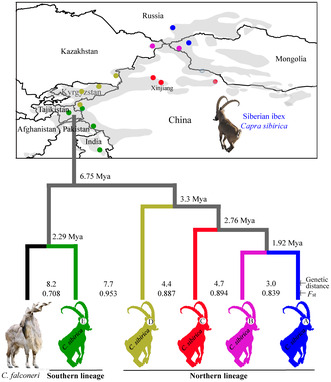

## 1 INTRODUCTION

The genus *Capra* inhabits restricted mountain areas in southern Europe, the Middle East, and Central Asia of the Palearctic region. It consists of 10 distinct species, such as wild goat/bezoar (*Capra aegagrus* Erxleben, 1777), domestic goat (*C. hircus*), markhor (*C. falconeri* Wagner, 1839), western tur (*C. caucasica* Güldenstädt & Pallas, 1783), eastern tur (*C. cylindricornis* Blyth, 1841), Iberian ibex (*C. pyrenaica* Schinz, 1838), Nubian ibex (*C. nubiana* Cuvier, 1825), Walia ibex (*C. walie* Rüppell, 1835), alpine ibex (*C. ibex* Linnaeus, 1758), and Siberian ibex (*C. sibirica* Pallas, 1776). They are found in areas from sea level to high mountainous habitats, some even at 6700 m above sea level in the Himalayas (Fedosenko & Blank, 2001). According to its skull, horn, external morphological characteristics, and phylogenetic position in a genetic tree, the Siberian ibex was considered a relic species of the genus *Capra* (Kazanskaya et al., 2007; Manceau et al., 1999; Pidancier et al., 2006; Reading & Shank, 2008). Based on fossil evidence, Pilgrim (2008) proposed that the *Capra* first appeared in Central Asia and diversified during the Plio‐Pleistocene. Gene introgression between ancestral bezoar and ibex types was likely occurred and may have contributed to the genetic makeup of now‐living *Capra* species (Pidancier et al., 2006).

**FIGURE 1:**
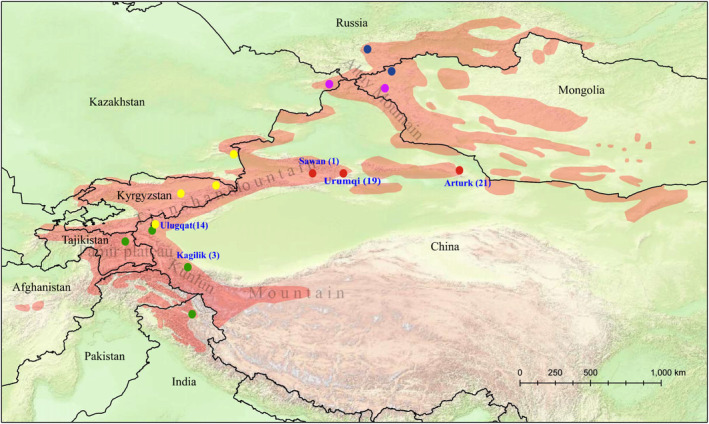
Distribution of Siberian ibex (*Capra sibirica*) in Central Asia (www.iucnredlist.org, downloaded on 23, March, 2022). The border lines are approximate. The round marks represent sampling sites from GenBank (out of Chinese territory) and this study (in Chinese territory). The different colors indicate different *C. sibirica* lineages: the blue, pink, yellow, red, and green correspond to clades A–E in the phylogenetic trees and networks, respectively. The numbers in parentheses represent the number of samples collected in this study.

The Siberian ibex, also known as Asiatic or Asian ibex, is the genus's largest and heaviest‐built species, with the longest horns on males (Fedosenko & Blank, 2001). It has the broadest distribution than any other *Capra* species, ranging from the Himalayas mountains in northern India and China through the Pamir plateau in eastern Pakistan, Afghanistan, Kyrgyzstan, Kazakhstan, and China to the Altai mountains in Mongolia, Russia, and China (Figure 1). This species is listed as “Near Threatened” both in the red list of IUCN and in the Red Book of Kyrgyzstan (Ingeborg et al., 2018; Reading et al., 2020), and one of the category II key protected wildlife in China (National Forestry and Grassland Administration, National Park Administration, 2021). Its population is decreasing due to trophy hunting in most of its range (Ingeborg et al., 2018), poaching, habitat degradation or fragmentation, conflicts with domestic animals, and natural diseases (Reading et al., 2020). As such, there are scarce genetic studies, especially in China.

*Capra sibirica* was once recognized as a subspecies of *C. ibex* (Abdukadir, 2006; Khan et al., 2016), but was later confirmed as a different species genetically and morphologically (Fedosenko & Blank, 2001; Kazanskaya et al., 2007; Pidancier et al., 2006). The intraspecific classification of this species is one of the most complex problems and is still controversial. According to the characteristics of body shape, coat color, and horns, Fedosenko and Blank (2001) and Wilson and Reeder (2005) classified *C. sibirica* into four subspecies, namely: those in the Altai mountains in Russia, Northwest China, Western Mongolia, and Northeastern Kazakhstan were regarded as *C. s. sibirica* Pallas, 1776; those in Central and Southern Mongolia and North China as *C. s. hagenbecki* Noak, 1903; those in the Tianshan mountains in Kyrgyzstan, Southeastern Kazakhstan, Tajikistan, North Afghanistan, and Western China as *C. s. alaiana* Noak, 1902; and those in North India and Pakistan as *C. s. sakeen* Blyth, 1842. Smith and Xie (2008) and Wang and Jie (2004) regarded *C. sibirica* in the Kunlun and Karakoram mountains as a separate subspecies, although some disagree with this classification (Abdukadir, 2003; Gao, 2005). Grubb et al. (2005), however, believed that *C. sibirica* should be defined as a monotypic species without dividing any subspecies. Topographic uplifts contribute to biodiversity evolution and have elevated speciation in mammals (Igea & Tanentzap, 2021). *C. sibirica* is a species of mountain dweller that inhabits the Altai, Tianshan, and Himalayan Mountains. These mountains are geographically remote and divided by the Junggar and Tarim basins. *C. sibirica* populations in the Altai and Himalaya mountains showed different external morphology (Sobanskiy, 1992). In light of these facts, we hypothesize that *C. sibirica* populations in different alpine habitats are likely genetically differentiated and may consist of several distinct populations.

Mitochondrial markers have been widely used in the phylogenetic studies of many taxa, including mammals (Abduriyim et al., 2022; Abduriyim, Nabi, & Halik, 2018; Abduriyim, Zibibulla, et al., 2018), and mitochondrial lineages were accepted as an important component of intra and interspecific biodiversity (Hu et al., 2021; Huang et al., 2023). Hence, to test our speculation, we analyzed mitochondrial cytochrome *b* (*cytb*) gene and control region (CR) sequences derived from feces, skin, or tissue samples collected in Xinjiang, China, together with sequences retrieved from GenBank. We aimed to elucidate the phylogeny and genetic structure of *C. sibirica* populations. Our findings are significant in terms of identifying conservation management units and providing effective protection.

## 2 MATERIALS AND METHODS

### 2.1 Sampling

In Xinjiang, 58 samples were collected, including 49 feces, six muscle, two skin, and one liver sample: 19 from Urumqi, 21 from Arturk, one from Sawan, 14 from Ulugqat, and three from Kagilik (Figure 1, Appendix 1). The tissue samples, including muscle, liver, and skin, were taken from individuals that died of natural causes or individuals that were poached. For fecal samples, we first observed *C. sibirica* individuals in the distance, took their positions, and only their fresh feces were collected after they had left to ensure the individual identity of fecal samples collected during field work. Identities were also determined based on relative stool and urine positions, as well as stool size and distances (Abduriyim, Nabi, & Halik, 2018; Abduriyim, Zibibulla, et al., 2018). All fecal and tissue samples were preserved in 96% ethanol at −80°C, while skin samples were frozen in plastic bags.

### 2.2 DNA extraction, amplification, and sequencing

The total genomic DNA of fecal samples was extracted using an Omega stool DNA extraction kit (D4015; Omega Bio‐tec), and that of muscle and skin samples was extracted using a Tiangen tissue/blood DNA extraction kit (DP328‐02; Tiangen), following the manufacturer's instructions. After electrophoresis detection, DNA concentration and purity were measured by the Thermo Nanodrop 1000 and stored at 4°C for later use.

The primer pairs of Cytb‐F (5′‐CATCGTTGTTATTCAACTACAAGAACACT‐3′) and Cytb‐R (5′‐ACCAGTATATTAATTGTACTACAAAGAC‐3′) for mtDNA *cytb* gene PCR amplification, CR‐F (5′‐ACCCACGCATACAAACACCC‐3′) and CR‐R (5′‐ACTGTGATGCTCG TGCCTAC‐3′) for CR gene were designed using Primer Premier v.5.0 software and were synthesized commercially. The PCR reactions were carried out in a total volume of 25 μL, containing 40–150 ng of DNA, 5 pmol of each primer, and 12.5 μL of Tiangen's 2× Taq PCR Master Mix, with a final volume of 25 μL adjusted with RNase‐Free dd‐water. The PCR thermal cycling conditions were as follows: pre‐denaturation at 94°C for 4 min, followed by 35 cycles of denaturation at 94°C for 30 s, annealing at 54°C (*cytb*)/60°C (CR) for 30 s, extension at 72°C for 45 s, and final extension at 72°C for 5 min. PCR products were run on 1% agarose gel electrophoresis to check relative purity and concentration and then were sent to Sangni Biotechnology (Shanghai) for sequencing. Raw sequences obtained were manually edited in SeqMan, aligned with MEGA v.6.0 (Tamura et al., 2013) using MUSCLE, and BLAST searched at NCBI to confirm their gene identity.

### 2.3 Data analysis

We used MEGA v.6.0 (Tamura et al., 2013) to align the obtained sequences, then used DnaSP v.5.10.01 (Librado & Rozas, 2009) to calculate the nucleotide composition, number of segregating sites (*S*), nucleotide diversity (*π*), haplotype diversity (*h*), pairwise population fixation indices (*F*_ST_), Fu's *F*_*S*_ and Tajima's *D*. The genetic distance was calculated using the Kimura two‐parameter model with 1000 bootstraps in MEGA v.6.0.

The phylogenetic analyses for *cytb* and *cytb* + CR sequences were performed in MEGA v.6.0 (Tamura et al., 2013) using the neighbor‐joining (NJ) method with the Kimura two‐parameter model and in IQ‐TREE v.1.6.8 (Minh et al., 2020) using the maximum likelihood algorithm (ML). Nodal supports for both the NJ and ML trees were calculated using bootstrapping with 1000 replications. Median‐joining network analyses were conducted separately for the different dataset in PopART v.1.7 (Leigh & Bryant, 2015). The optimal model for Bayesian inference (BI) was chosen based on the Bayesian Information Criterion (BIC) by using Kakusan v.4 (Tanabe, 2011). The BI phylogenetic trees were constructed in MrBayes v.3.1.2 (Ronquist & Huelsenbeck, 2003). Two independent runs were performed for 2 million generations each, with Markov chains sampled every 100 generations. The convergence of parameter values for two runs was checked in Tracer v.1.7.2 (Rambaut et al., 2018), prior to discarding the first 25% trees as burn‐in.

Divergence time estimation was performed based on mtDNA *cytb* sequences (376, 679, and 1095 bp) of all *Capra* species (*C. aegagrus*, DQ514541; *C. hircus*, D84201; *C. cylindricornis*, DQ246769; *C. caucasica*, AF034738; *C. falconeri*, AF034736 and D84202; *C. walie*, OW568914; *C. nubiana*, NC020624; *C. ibex*, NC020623; *C. pyrenaica*, NC020625), Himalayan tahr (*Hemitragus jemlahicus*, NC020628), Bharal (*Pseudois nayaur*, AF398362), Tibetan antelope (*Pantholops hodgsonii*, NC007441), and Gemsbok (*Oryx gazella*, NC016422). The sequences were aligned by MUSCLE in MEGA v.6.0 (Edgar, 2004; Tamura et al., 2013). The nucleotide substitution model was selected based on the BIC by using jModeltest v.2.1.10 (Darriba et al., 2012). To estimate the divergence times of the *Capra* species, Bayesian inference was performed in BEAST v.2.6.7 (Bouckaert et al., 2019). The best selected model, HKY with gamma site model, was used for the site model. The strict clock model and Yule model were used. The molecular clock was calibrated using a fossil prior as a softbound, all *Capra* species that share a more recent common ancestor with it than with *Panthlops hodgsonii*, calibration point stem Caprini (Minimum age: 8.9 Mya; Lognormal, 8.9–12.9 Mya (*x* = 10.8 Mya, log (SD) = 0.095, *M* = 10.8 Mya); Alcalá & Morales, 1997; Bibi, 2013; Dam et al., 2001). Two independent Markov Chain Monte Carlo (MCMC) analyses were run for 10 million generations, with parameters sampled every 2000 generations. The first 10% trees were discarded as a burn‐in from each MCMC run. MCMC chain convergence of all parameters of interest and ESS > 200 for each of the tree runs were confirmed using Tracer v.1.7.2 (Rambaut et al., 2018). TreeAnnotator v.2.6.7 was used to summarize the representative summary tree with the mean ages of all the nodes and the corresponding 95% highest posterior density (HPD) ranges and to calculate posterior clade probability for each node (Bouckaert et al., 2019). The maximum clade credibility tree visualization and geological timescale were plotted using the R packages ggtree v.3.2.1 and deeptime v.0.2.2 in R v.4.1.2 (Gearty, 2022; R Core Team, 2021; Yu, 2020).

## 3 RESULTS

### 3.1 Sequence variation

Since the reliability of the beginning and ending sequence reads was very low, the final dataset of 54 *cytb* sequences we obtained was 1095 bp long, and the final dataset of 43 CR sequences was 935 bp long, with indels in some sequences. PCR amplification from a few samples failed (Appendix 1), probably owing to the longer storage time or low quality of the sample. Because sample size or haplotype number was limited for some localities (e.g., one sample from Sawan; one haplotype in the *cytb* gene from Urumqi, Kagilik, and Ulugqat‐f; and one haplotype in the CR gene from Ulugqat‐s), some genetic parameters were not calculated. Due to the fact that the sequences from Ulugqat were split into two distinct lineages in phylogenetic trees and networks, we calculated genetic parameters accordingly, namely separated into two groups (Ulugqat‐s is grouped with *C. sibirica*, Ulugqat‐f with *C. falconeri*) in Table 1. We identified 10 different haplotypes with 127 segregating sites in 54 *cytb* sequences and 20 haplotypes with 168 segregating sites in 43 CR sequences (Table 1). The Arturk population had higher haplotype diversity and nucleotide diversity in both *cytb* and CR genes than other populations (Table 1). The values of Fu's *F*_S_ and Tajima's *D* of the Siberian ibex population were both positive, indicating that the *C. sibirica* population is shrinking and the rare alleles in the population exist at low frequency (Table 1).

**TABLE 1:** Genetic diversity parameters of mitochondrial *cytb* (1095 bp) and control region (935 bp) sequences for geographic populations of *Capra sibirica* from Xinjiang, China.

Location	Gene	*n*	Hn	*S*	*h*	*π*	Fu's *F*_S_	Tajima's *D*
Urumqi	*Cytb*	18	1	–	–	–	–	–
	CR	12	4	33	0.7308	0.0132	−1.2408	−0.5084
Arturk	*Cytb*	18	4	18	0.7135	0.0033	−1.8788	−1.1796
	CR	14	8	31	0.9121	0.0175	1.6185**	1.2452
Ulugqat‐s	*Cytb*	6	2	1	0.3333	0.0003	−0.9502	−0.9330
	CR	6	1	–	–	–	–	–
Ulugqat‐f	*Cytb*	8	1	–	–	–	–	–
	CR	7	4	10	0.8095	0.0047	−1.1216	−1.0489
Kagilik	*Cytb*	3	1	–	–	–	–	–
	CR	3	3	9	1.0000	0.0084	–	–
Sawan	*Cytb*	1	1	–	–	–	–	–
	CR	1	1	–	–	–	–	–
Total	*Cytb*	54	10	127	0.8302	0.0366	1.7982**	1.5464
	CR	43	21	168	0.9468	0.0832	1.5632*	1.6060

*Note*: The *n*, Hn, *S*, *h*, and *π* signify the number of samples, number of haplotypes, segregation sites, haplotype diversity, and nucleotide diversity, respectively. – signifies no difference between sequences or insufficient data to calculate, *p < .05, **p < .01.

### 3.2 Phylogenetic relationships

**FIGURE 2:**
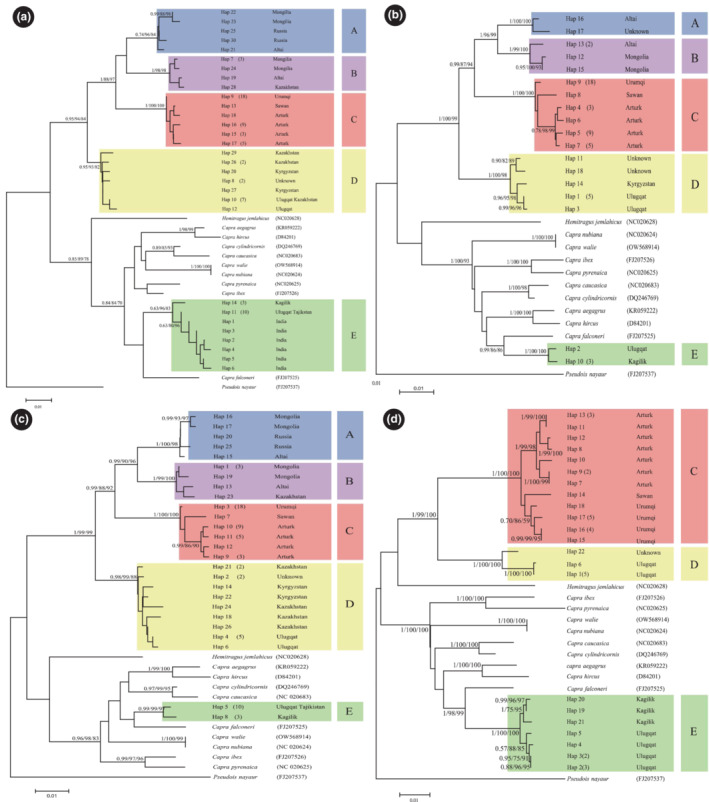
Phylogenetic relationship of *Capra sibirica* populations was constructed using Bayesian inference (BI), maximum likelihood (ML), and neighbor‐joining (NJ) methods based on the mtDNA cytochrome *b* gene of the 376 bp dataset (a), 1095 bp dataset (b), 679 bp dataset (c), and concatenated sequences of *cytb* and the control region (d). The trees rooted with *Pseudois nayaur* were bootstrapped with 1000 replicates in ML and NJ, and nodal supports of ≥80 (ML and NJ) and 0.7 (BI) were given. Haplotypic sequences for the *C. sibirica* species were used, and the numbers in the brackets next to them indicate the number of sequences of that particular haplotype. The GenBank accession numbers for the rest of the *Capra* species were provided on the right of their Latin names within brackets.

**FIGURE 3:**
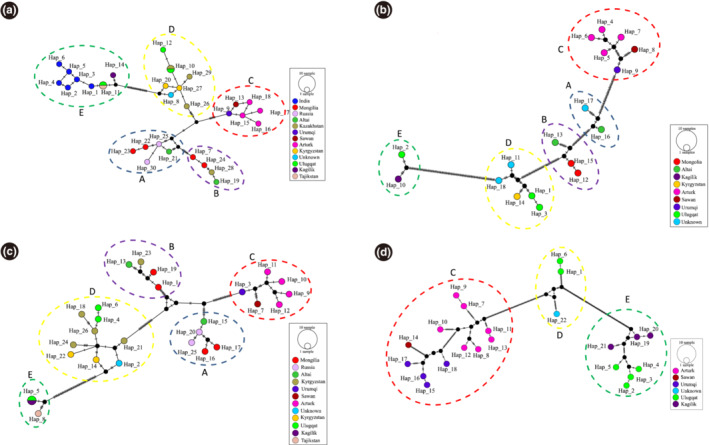
Network analysis showing the relationships of the mtDNA cytochrome *b* sequence of the 376 bp dataset (a), 1095 bp dataset (b), 679 bp dataset (c), and concatenated sequences of *cytb* and the control region (d) of *C. sibirica*. The grouping pattern of sequences was in line with the phylogenetic trees in Figure 2. The colored circles (A–E) corresponded to clades A–E in the phylogenetic tree.

In the phylogenetic analyses, we also included *cytb* sequences of *C. sibirica* individuals from Mongolia, Russia, Kazakhstan, Kirgizstan, Tajikistan, and India (Appendix 1), and *cytb* + CR (complete mitochondrial genome) sequences of other *Capra* species (their accession numbers were provided in the phylogenetic trees) retrieved from NCBI GenBank. Because the lengths of sequence from NCBI were different and to include more sequences from different locations, phylogenetic trees were constructed based on *cytb* gene datasets of 376, 679, and 1095 bp, respectively, using the Bharal (*Pseudois nayaur*) sequence as an outgroup. As a result, all trees estimated using ML and NJ inferences and different datasets yielded highly congruent topologies (Figure 2). The phylogenetic tree and haplotype network constructed using *cytb* + CR sequences were also congruent with the topologies of *cytb* sequences (Figures 2 and 3). Surprisingly, *C. sibirica* sequences clustered into five distinct monophyletic clades, named A, B, C, D, and E. Of these, clades A, B, C, and D formed a monophyletic *C. sibirica* Northern lineage, separating from other *Capra* sequences. Within Northern lineage, clade A is composed of sequences from Russia, Mongolia, and Altai (Russia), clade B from Mongolia, Kazakhstan, and Altai (Russia), while clade C contained sequences from Urumqi, Sawan and Arturk and clade D from Ulugqat, Kyrgyzstan, and Kazakhstan. Intriguingly, more than half of the sequences from Ulugqat and sequences from Kagilik, Tajikistan, and India formed another distinct clade E, the Southern lineage, a monophyletic sister lineage to Markhor (*C. falconeri*). The clustering pattern of haplotype sequences in network analyses was consistent with the phylogenetic topology (Figure 2). The genetic distance between the five clades ranged from 3.0% to 8.6%. The maximum value within the Northern lineage was between clades B and C (4.7%), while the minimum value (3.0%) was between clades A and B (Table 2). The *F*_ST_ values between the five clades ranged from 0.839 to 0.960. The maximum value within the Northern lineage was between clades B and C (0.894), while the minimum value (0.839) was between clades A and B (Table 2).

**TABLE 2:** Genetic distance (below the diagonal) and *F*_ST_ (above the diagonal) between clades A and E calculated based on *cytb* (679 bp) sequences.

	A	B	C	D	E
A		0.839	0.889	0.887	0.960
B	3.0		0.894	0.882	0.958
C	4.3	4.7		0.887	0.956
D	4.3	4.2	4.4		0.953
E	8.6	8.6	8.1	7.7	

*Note*: Genetic distance is expressed as a percentage.

### 3.3 Divergence time estimations

**FIGURE 4:**
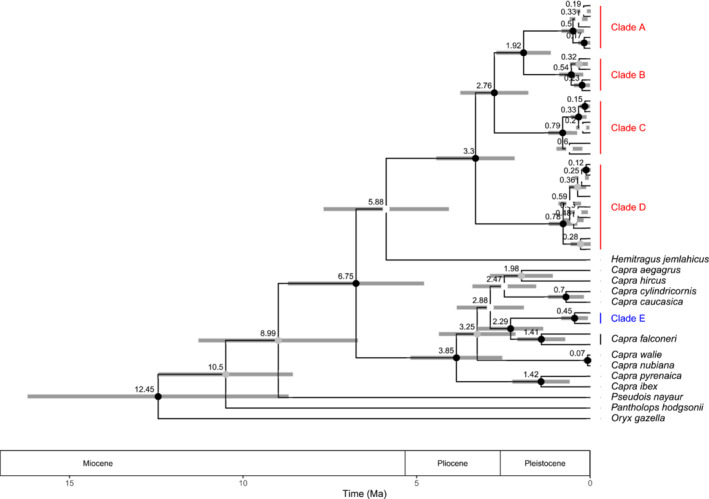
Chronogram for Caprinies. The mean (numbers) and 95% highest posterior density (HPD, gray sticks) of individual node ages are shown at selected branches. Letters (A–E) next to *Capra* clades refer to intraspecific phylogenetic haplogroups.

The divergence times of *Capra* species were inferred based on mtDNA *cytb* 679 bp sequences using BEAST2 (Figure 4, Table 3). The mean time to the most common ancestor (TMRCA) of all *Capra* species was 6.75 million years ago (Mya; 95%HPD: 4.88–8.80) in the Late Miocene epoch. The mean TMRCA of the Northern lineages (clades A–D) of *C. sibirica* was 3.30 Mya (95%HPD: 2.21–4.46) in the Late Pliocene epoch. The mean TMRCA of the Southern lineage (Clade E) of *C. sibirica* and *C. falconeri* was 2.29 Mya (95%HPD: 1.42–3.29) in the Early Pleistocene epoch. Inferred divergence times from 1095 bp *cytb* sequences were comparable to those from 679 bp *cytb* sequences, although estimates from 376 bp *cytb* sequences were marginally higher than those from the other two datasets (Table 3).

**TABLE 3:** Divergence time (Mya) estimates of *Capra* species inferred using BEAST2 based on three different mtDNA *cytb* dataset.

Dataset (bp)	*Capra* species		Clade E and *Capra falconeri*		Clade E (Mya)		Clades A–D (Mya)	
	Mean	95%HPD	Mean	95%HPD	Mean	95%HPD	Mean	95%HPD
376	10.49	8.59–12.42	3.33	1.75–5.01	1.15	0.48–1.93	3.85	2.26–5.60
679	6.75	4.88–8.80	2.29	1.42–3.29	0.45	0.99–0.87	3.30	2.21–4.46
1095	6.19	4.65–7.84	2.05	1.40–2.73	0.41	0.19–0.57	3.13	2.20–4.07

## 4 DISCUSSION

Identifying valid species/evolutionary significant units and/or management units is a priority for effective conservation measurements and policies and plays a pivotal role in systematics and evolutionary biology. We found two major lineages in the phylogenetic tree and network analyses of *cytb* and CR gene sequences (Figures 2 and 3), one containing sequences from the northern range (clades A–D) that are sister lineages to other *Capra* species and the other containing sequences from the southern range that are sister to *C. falconeri*, indicating paraphyly of *C. sibirica*. The monophyletic separation of the Northern lineage from other *Capra* species is in line with the previous studies (Joshi et al., 2020; Kazanskaya et al., 2007; Pidancier et al., 2006; Zheng et al., 2020). Moreover, the genetic distances between clades A and E (Table 2) were greater than those between *Capra* species (Kazanskaya et al., 2007), and they were highly structured and divergent genetically. The divergence time of these clades' dates back much earlier (3.30–1.92 Mya). The grouping pattern of clades within the Northern lineage corresponds to their geographic origins. Based on the traffic‐light system for designating taxonomic certainty in mammals (Kitchener et al., 2017, 2022), it is highly likely that these clades represent distinct species. Different geographical populations' coat colors and body sizes support this notion (Fedosenko & Blank, 2001), and the nature of species' altitudinal migration may cause significant genetic differentiation of populations (Reading et al., 2020; Sobanskiy, 1992; Xu, 1995). Likewise, five different species of *Ovis ammon* were identified in different mountains in Northwestern China (Jiang et al., 2016), where they are sympatric to Clades A–D, respectively. However, the morphological and biogeographical data, including modeling of species distributions based on habitat usage, for example, *Capra walie* (Gebremedhin et al., 2009), will be helpful in confirming whether populations are valid species.

Southern lineage (Clade E) clustering with *C. falconeri* is consistent with Y‐chromosomal and genomic phylogenies of *C. falconeri* and *C. sibirica* (Zheng et al., 2020), but not with Y‐chromosomal AMELY and ZFY gene phylogenies (Pidancier et al., 2006). Hybridization and/or incomplete lineage sorting (ILS) may explain this phenomenon. Many studies on adaptive introgression in mammals may be useful as references for our study (Chen et al., 2018; Hedrick, 2013; Hu et al., 2019; Miao et al., 2017; Wu et al., 2018). According to the topology of Whole‐Genome Sequence (WGS) and Y‐DNA trees (Zheng et al., 2020), geographically neighboring *Capra* species are closely related to each other, for example, *C. sibirica* and *C. falconeri*, suggesting the male‐biased dispersal of *Capra* species after speciation of the TMRCA of *Capra*. After the speciation between *C. falconeri* and *C. sibirica* at about 6.75 Mya, hybridization of the two species frequently occurred, probably male of *C. falconeri* crossbred with female of *C. sibirica*, so there was no mtDNA introgression at this time. This caused a large portion of autosomal DNA and Y‐DNA from two species to become closely related to each other. Then, the mtDNA introgression from *C. falconeri* into only the southern population of *C. sibirica* (Clade E) occurred in the early Pleistocene epoch (Figure 4). The phylogenetic anomalies among closely related bovine species were explained by ILS rather than hybridization, and the Southern lineage being sister to other *Capra* species seems to be due to ILS. However, given that the clustering pattern of the Northern lineage and *C. falconeri* in our mtDNA trees (Figures 2 and 3) was in line with the topology of the WGS and Y chromosome (Zheng et al., 2020), and the divergence time between lineages was much earlier (Table 3), we cannot exclude the possibility of hybridization or introgression between these two species, owing to their geographic proximity, as reported in other sympatric bovine species (Fadakar et al., 2020). Thus, population genomic studies with dense geographical sampling are needed to unveil this phenomenon, as sufficiently many unlinked genes/loci always return the correct topology (Mossel & Roch, 2010).

In China, the government has taken effective measures, such as hunting bans and the establishment of protected areas, to protect wild animals for decades. The population of *C. sibirica* has been continuously increasing, and it has recently been downgraded to National Category II from Category I (National Forestry and Grassland Administration, National Park Administration, 2021, http://www.forestry.gov.cn). This species is mainly distributed in the Xinjiang, Inner Mongolia, Tibet, and Gansu provinces of China. In our analyses, samples were collected from five different geographic locations, clustering into three clades, namely clades C, D, and E. Samples from Urumqi, Sawan, and Arturk formed a distinct clade C (Figure 2). In the network analysis, haplotypes 15–18 from Arturk were connected to other populations through haplotype 9 from Urumqi (Figure 3a), indicating that they might disperse from Urumqi populations to the eastern edges of Tianshan Mountain. Samples from Ulugqat were split into two clades D and E with notable genetic differentiation (Table 2), indicating that Ulugqat and its vicinities are the contact zone of two clades. The denser and wider sampling in the Pamir plateau and Tianshan Mountain will help to identify wider contact regions, which helps to propose conservation measurements and policies related to this species. Taking our results altogether, one can assume that sampling from other distributional regions, say Altai Mountain in Xinjiang, Inner Mongolia, Gansu, and Qinghai provinces, will likely identify more distinct populations within Chinese territory.

Our divergence time estimation results for Caprinies (Figure 4, Table 3) were similar to those of Ropiquet and Hassanin (2005) and Bibi (2013), suggesting that the *Capra* diversification after adaptive radiation of the tribe Caprini during the late Miocene (Gebremedhin et al., 2009) followed major climatic shifts that occurred during the late Miocene, Pliocene, and Pleistocene, similar to other terrestrial mammals (Ge et al., 2013). We deduced that radiation of the Northern lineage occurred concurrently with that of the other *Capra* species, resulting in the diversification of clades A–D, Clade E, and *C. falconeri* between 3.30 and 1.92 Mya. This is in agreement with the large number of species in several subfamilies that originated during this period, in which the diversification of C4 plants accelerated (Christin et al., 2009) and was followed by the replacement of a large area of forest on the earth by open grassland in “Nature's Green Revolution” (Osborne & Beerling, 2006). The subsequent tectonic changes in the Pamir and Tibetan plateaus (Cao et al., 2013; Thompson et al., 2015) and glacial changes (Ge et al., 2013) likely facilitated the radiation as well. During the late Pliocene and Pleistocene glacial periods (2.77–1.99 Ma), this may have allowed the subsequent colonization of clade C to Tianshan Mountains and clades A and B to Altai, respectively (Cook et al., 2017)

## AUTHOR CONTRIBUTIONS

**Rui‐Rui Wang:** Formal analysis (equal); investigation (equal); validation (equal); visualization (equal); writing – original draft (supporting). **Pei‐Pei Dong:** Formal analysis (equal); investigation (equal); validation (equal); visualization (equal). **Daisuke Hirata:** Data curation (equal); formal analysis (equal); methodology (equal); validation (equal); visualization (equal); writing – original draft (supporting); writing – review and editing (equal). **Shamshidin Abduriyim:** Conceptualization (lead); data curation (equal); formal analysis (supporting); funding acquisition (lead); investigation (equal); methodology (equal); project administration (lead); resources (lead); supervision (lead); validation (supporting); visualization (supporting); writing – original draft (lead); writing – review and editing (equal).

## FUNDING INFORMATION

This study was funded by the National Natural Science Foundation of China (No. 32260328).

## CONFLICT OF INTEREST STATEMENT

The authors declare no conflict of interest.

## Data Availability

The mtDNA sequences we obtained have been deposited in the DDBJ databases under accession numbers LC706311–LC706364 for *cytb* and LC711033–LC711075 for CR.

## APPENDIX 1 Profile of samples and GenBank sequences (of *Capra sibirica*) analysed in this study.

### 1.1

**Table:**

No.	Sample ID	Location	Sample type	Figure 2a haplotype	Figure 2b haplotype	Figure 2c haplotype	Figure 2d haplotype	Accession number	
								*Cytb*	CR
1	capra_sib_nj01	Ulugqat	Feces	Hap_10	Hap_1	Hap_4	Hap_5	LC706333	LC711033
2	capra_sib_nj02	Ulugqat	Feces	Hap_10	Hap_1	Hap_4	Hap_5	LC706329	LC711034
3	capra_sib_nj03	Ulugqat	Feces	Hap_10	Hap_1	Hap_4	Hap_5	LC706336	LC711035
4	capra_sib_nj04	Ulugqat	Feces	Hap_10	Hap_1	Hap_4	Hap_5	LC706337	LC711036
5	capsib_njP2	Ulugqat	Skin	Hap_10	Hap_1	Hap_4	Hap_5	LC706341	LC711037
6	capsib_njP3	Ulugqat	Skin	Hap_12	Hap_3	Hap_6	Hap_10	LC706342	LC711038
7	capsib_nj5	Ulugqat	Feces	Hap_11	Hap_2	Hap_5	Hap_6	LC706338	LC711039
8	capsib_nj6	Ulugqat	Feces	Hap_11	Hap_2	Hap_5	–	LC706330	–
9	capsib_nj7	Ulugqat	Feces	Hap_11	Hap_2	Hap_5	Hap_6	LC706331	LC711040
10	capsib_nj8	Ulugqat	Feces	Hap_11	Hap_2	Hap_5	Hap_7	LC706332	LC711041
11	capsib_nj9	Ulugqat	Feces	Hap_11	Hap_2	Hap_5	Hap_7	LC706339	LC711042
12	capsib_nj10	Ulugqat	Feces	Hap_11	Hap_2	Hap_5	Hap_6	LC706334	LC711043
13	capsib_nj11	Ulugqat	Feces	Hap_11	Hap_2	Hap_5	Hap_8	LC706335	LC711044
14	capsib_njJ1	Ulugqat	Muscle	Hap_11	Hap_2	Hap_5	Hap_9	LC706340	LC711045
15	capsib_YC1	Kagilik	Muscle	Hap_14	Hap_10	Hap_8	Hap_11	LC706344	LC711046
16	capsib_YC2	Kagilik	Muscle	Hap_14	Hap_10	Hap_8	Hap_12	LC706345	LC711047
17	capsib_YC3	Kagilik	Muscle	Hap_14	Hap_10	Hap_8	Hap_13	LC706346	LC711048
18	capsib_hx1	Urumqi	Feces	Hap_9	Hap_9	Hap_3	Hap_14	LC706312	LC711049
19	capsib_hx2	Urumqi	Feces	Hap_9	Hap_9	Hap_3	Hap_3	LC706321	LC711050
20	capsib_hx6	Urumqi	Feces	Hap_9	Hap_9	Hap_3	Hap_4	LC706325	LC711051
21	capsib_hx7	Urumqi	Feces	Hap_9	Hap_9	Hap_3	Hap_15	LC706326	LC711052
22	capsib_hx8	Urumqi	Feces	Hap_9	Hap_9	Hap_3	Hap_4	LC706327	LC711053
23	capsib_hx9	Urumqi	Feces	Hap_9	Hap_9	Hap_3	–	LC706328	–
24	capsib_hx10	Urumqi	Feces	Hap_9	Hap_9	Hap_3	Hap_3	LC706313	LC711054
25	capsib_hx11	Urumqi	Feces	Hap_9	Hap_9	Hap_3	Hap_4	LC706314	LC711055
26	capsib_hx12	Urumqi	Feces	Hap_9	Hap_9	Hap_3	Hap_3	LC706315	LC711056
27	capsib_hx13	Urumqi	Feces	Hap_9	Hap_9	Hap_3	Hap_4	LC706316	LC711057
28	capsib_hx14	Urumqi	Feces	Hap_9	Hap_9	Hap_3	Hap_4	LC706317	LC711058
29	capsib_hx16	Urumqi	Feces	Hap_9	Hap_9	Hap_3	Hap_3	LC706318	LC711060
30	capsib_hx17	Urumqi	Feces	Hap_9	Hap_9	Hap_3	–	LC706319	–
31	capsib_hx19	Urumqi	Feces	Hap_9	Hap_9	Hap_3	–	LC706320	–
32	capsib_hx20	Urumqi	Feces	Hap_9	Hap_9	Hap_3	–	LC706322	–
33	capsib_hx21	Urumqi	Feces	Hap_9	Hap_9	Hap_3	–	LC706323	–
34	capsib_hx22	Urumqi	Feces	Hap_9	Hap_9	Hap_3	–	LC706324	–
35	capsib_hx4M	Urumqi	Muscle	Hap_9	Hap_9	Hap_3	–	LC706311	–
36	capsib_SLG	Sawan	Muscle	Hap_13	Hap_8	Hap_7	Hap_1	LC706343	LC711061
37	capsib_yw_a	Arturk	Feces	Hap_15	Hap_4	Hap_9	Hap_16	LC706347	LC711062
38	capsib_yw_b	Arturk	Feces	Hap_16	Hap_5	Hap_10	–	LC706348	–
39	capsib_yw_c	Arturk	Feces	Hap_17	Hap_7	Hap_11	Hap_17	LC706349	LC711063
40	capsib_YWe	Arturk	Feces	Hap_16	Hap_5	Hap_10	Hap_2	LC706350	LC711065
41	capsib_ywA	Arturk	Feces	Hap_16	Hap_5	Hap_10	Hap_18	LC706357	LC711066
42	capsib_ywB	Arturk	Feces	Hap_17	Hap_7	Hap_11	Hap_19	LC706358	LC711067
43	capsib_ywC	Arturk	Feces	Hap_16	Hap_5	Hap_10	Hap_2	LC706359	LC711068
44	capsib_ywE	Arturk	Feces	Hap_17	Hap_7	Hap_11	Hap_19	LC706360	LC711070
45	capsib_ywF	Arturk	Feces	Hap_17	Hap_7	Hap_11	Hap_19	LC706361	LC711071
46	capsib_ywx1	Arturk	Feces	Hap_15	Hap_4	Hap_9	Hap_20	LC706362	LC711072
47	capsib_ywx2	Arturk	Feces	Hap_18	Hap_6	Hap_12	Hap_21	LC706363	LC711073
48	capsib_ywx3	Arturk	Feces	Hap_15	Hap_4	Hap_9	Hap_20	LC706364	LC711074
49	capsib_yw_f	Arturk	Feces	Hap_16	Hap_5	Hap_10	–	LC706351	–
50	capsib_yw_g	Arturk	Feces	Hap_16	Hap_5	Hap_10	–	LC706352	–
51	capsib_yw_i	Arturk	Feces	Hap_16	Hap_5	Hap_10	–	LC706353	–
52	capsib_yw_j	Arturk	Feces	Hap_16	Hap_5	Hap_10	–	LC706354	–
53	capsib_yw_k	Arturk	Feces	Hap_16	Hap_5	Hap_10	–	LC706355	–
54	capsib_yw7	Arturk	Liver	Hap_17	Hap_7	Hap_11	–	LC706356	–
55	capsib_ywx4	Arturk	Feces	–	–	–	–	–	LC711075
56	capsib_ywD	Arturk	Feces	–	–	–	–	–	LC711069
57	capsib_ywd	Arturk	Feces	–	–	–	–	–	LC711064
58	capsib_hx15	Urumqi	Feces	–	–	–	–	–	LC711059
59	CisIB10	Mongolia	Unknown	Hap_7	–	Hap_7	–	DQ514550	–
60	41s	Altai	Unknown	Hap_7	Hap_15	Hap_1	–	DQ246774	–
61	G1253	Mongolia	Unknown	Hap_7	Hap_12	Hap_1	–	AB743826	–
62	9	Mongolia	Unknown	Hap_24	–	Hap_19	–	KT260194	–
63	15s	Altai	Unknown	Hap_19	Hap_13	Hap_13	–	DQ246771	–
64	74Cs	Kazakhstan	Unknown	Hap_28	–	Hap_23	–	KX096851	–
65	15	Russia	Unknown	Hap_25	–	Hap_20	–	KT283242	–
66	43s	Altai	Unknown	Hap_21	Hap_16	Hap_15	–	DQ246775	–
67	76Cs	Russia	Unknown	Hap_30	–	Hap_25	–	KX096853	–
68	CisIB2	Mongolia	Unknown	Hap_22	–	Hap_16	–	DQ514551	–
69	Cs01	Mongolia	Unknown	Hap_23	Hap_17	Hap_17	–	KF990328	–
70	–	Unknown	Unknown	Hap_8	Hap_18	Hap_2	Hap_22	NC020626	–
71	–	Unknown	Unknown	Hap_8	Hap_11	Hap_2	–	AF034734	–
72	26	Kazakhstan	Unknown	Hap_26	–	Hap_21	–	KT283245	–
73	3	Kazakhstan	Unknown	Hap_26	–	Hap_21	–	KT633870	–
74	25	Kyrgyzstan	Unknown	Hap_27	–	Hap_22	–	KT633871	–
75	75Cs	Kazakhstan	Unknown	Hap_29	–	Hap_24	–	KX096852	–
76	29s	Kyrgyzstan	Unknown	Hap_20	Hap_14	Hap_14	–	DQ246772	–
77	4	Kazakhstan	Unknown	Hap_10	–	Hap_18	–	KT246294	–
78	82Cs	Kazakhstan	Unknown	Hap_10	–	Hap_26	–	KY366503	–
79	20s	Tajikistan	Unknown	Hap_11	Hap_2	Hap_5	–	DQ246800	–
80	161Cs	Tajikistan	Unknown	Hap_11	–	Hap_5	–	KY996521	–
81	16s	Altai	Unknown	Hap_19	Hap_13	Hap_13	–	DQ246779	–
82	MT2494	India	Unknown	Hap_1	–	–	–	OK315797	–
83	MT2415	India	Unknown	Hap_2	–	–	–	OK315798	–
84	MT2414	India	Unknown	Hap_3	–	–	–	OK315799	–
85	MT2416	India	Unknown	Hap_4	–	–	–	OK315800	–
86	MT3410	India	Unknown	Hap_5	–	–	–	OK315801	–
87	MT2418	India	Unknown	Hap_6	–	–	–	OK315802	–

*Note*: The GenBank accession numbers (or samples) from line number 1 to line number 58 were obtained in this study, the rest of which were downloaded from GenBank. “–” indicates not applicable.
